# Compassion in nursing: Solution or stereotype?

**DOI:** 10.1111/nin.12271

**Published:** 2018-12-11

**Authors:** Stephanie Tierney, Roberta Bivins, Kate Seers

**Affiliations:** ^1^ Nuffield Department of Primary Care Health Sciences University of Oxford Oxford UK; ^2^ Department of History University of Warwick Coventry UK; ^3^ Warwick Research in Nursing Warwick Medical School, University of Warwick Coventry UK

**Keywords:** compassionate care, healthcare culture, history, leadership, nursing, policy

## Abstract

Compassion in healthcare has received significant attention recently, on an international scale, with concern raised about its absence during clinical interactions. As a concept, compassionate care has been linked to nursing. We examined historical discourse on this topic, to understand and situate current debates on compassionate care as a hallmark of high‐quality services. Documents we looked at illustrated how responsibility for delivering compassionate care cannot be consigned to individual nurses. Health professionals must have the right environmental circumstances to be able to provide and engage in compassionate interactions with patients and their relatives. Hence, although compassionate care has been presented as a straightforward solution when crisis faces health services, this discourse, especially in policy documents, has often failed to acknowledge the system‐level issues associated with its provision. This has resulted in simplistic presentations of ‘compassion’ as inexpensive and the responsibility of individual nurses, a misleading proposal that risks devaluing the energy and resources required to deliver compassionate care. It also overlooks the need for organisations, not just individuals, to be charged with upholding its provision.

## 1 INTRODUCTION

‘Compassionate care’ is a term that has entered the public consciousness over recent years. It lacks a clear, agreed meaning (Dewar & Nolan, 2013), although recurring elements are present across different definitions. For example, articles often mention its relational nature; it involves identifying and responding to what is important to patients (Dewar, Adamson, Smith, Surfleet, & King, 2014), and treating them as unique individuals (Wiklund, Gustin, & Wagner, 2013). Another common theme in definitions is its association with suffering (Burnell & Agan, 2013; Van der Cingel, 2009); compassion implies a deep connection with *and* motivation to alleviate someone's suffering (von Dietze & Orbb, 2000; Wiklund et al., 2013^2013^). According to Gilbert (2013), key attributes required for this to transpire are (a) sympathy (being able to feel for others), (b) empathy (being able to understand how others are feeling), (c) distress tolerance (letting things be, accepting emotions), (d) sensitivity to distress (picking up and noticing needs of self and others), (e) being non‐judgemental (not condemning others) and (f) care for well‐being (having motivation to support and help). Debate has been expressed about the possibility of augmenting such innate qualities, which may blossom or wither depending on the setting in which people work (Schwartz, 1995); hence, it is argued that environmental factors need to be conducive to accessing and realising attributes associated with compassionate care (Tierney, Seers, Tutton, & Reeve, 2017). This will be explored in more detail below.

The rise in debate and discussion of compassion in health services, over recent years, has occurred following examples of poor care. In the UK, the most prominent of these centred on the exposure of serious failings at Mid‐Staffordshire NHS Foundation Trust Hospital in England. Two inquiries, first private and then public (both chaired by independent barrister Robert Francis), sparked a continuing discussion about ‘quality of care’ in the NHS. The resulting report of the public inquiry (Francis, 2013) outlines what took place within this organisation and included 290 recommendations to promote more compassionate care, under stronger leadership, in a system that facilitates openness, transparency and candour. It gained international attention by testifying that basic elements of care were often overlooked, including patients being left in soiled beds, having water placed out of reach and receiving inadequate support for activities such as feeding (Francis, 2013). Within the report, there is a plea to put patients first in all aspects of healthcare and for greater compassion in their treatment. These calls, particularly in relation to care in hospitals and other residential institutions, have been loudest in relation to nursing. Indeed, while underfunding, understaffing, poor management, bullying and a host of other factors clearly played a role in the actual and perceived deterioration of bedside care, it was patient and family assertions of ‘callousness’ and targets‐driven gamesmanship among nurses and nurse–managers at Mid‐Staffordshire that provoked the most intense public and professional debate.

This discussion paper draws on historical precedents to explore the past, present and potential future of compassion as a hallmark of high‐quality professional nursing. We use these documents to illustrate why and when compassion has been mobilised by nurses, policy‐makers and others as a response to conditions within the NHS; we will consider the impacts, both positive and negative, of previous peaks in compassion and caring discourse on professional identities, practices and standards; and we will suggest ways in which the often highly emotive and politicised subject of compassion in nursing can still speak to the challenges faced by nurses and the NHS today.

## 2 HISTORICAL SYNOPSIS ON THE POLITICS AND ECONOMICS OF ‘COMPASSION’ IN NURSING CARE

As historians have documented, highly gendered understandings of care—almost invariably framed in moral and emotional as well as technical terms—have been central to the emergence of the nursing profession, since Charles Dickens caricatured the untrained and uncaring nurse through the drunken and immoral figure of Sarah Gamp (Abel‐Smith, 1960; Rafferty, 1996; Reverby, 1987). Indeed, visions of nursing as a vocation steeped in caring, sympathy and selflessness were at the heart of its transition from trade to profession in the late 19th century. This historical transition remains a frequent reference point in professional discussions around compassionate care and its relationship to nurse professionalism (Antrobus, 1997; Bradshaw, 2011; Kyle, 1995). As historians and sociologists of nursing have subsequently written, this positioning of the professional nurse has had profound impacts on public expectations, policy formulation (especially in relation to pay) and nursing practices ever since (Bessant, 1992).

Compassion could be regarded as today's buzzword for the same complexity of empathy, emotional labour and self‐sacrifice that linked nursing to religion and moral virtues during its professionalisation (Sellman, 1997). Compassion underpinned Florence Nightingale's depiction of the nursing character, and, in texts on nursing that followed, words associated with compassion (rather than this term itself) were employed—like virtuous, loving, kind, unselfish, gentle‐hearted (Bradshaw, 2011). A search of the literature suggests the phrase ‘compassionate care’ was first used during the 1970s in relation to nursing (Lanara, 1976), highlighting its conceptual background in the older, enduring values referred to above. After this, in the 1980s, it was often used in writing around caring for people with HIV/AIDS; hence, compassion was linked, at this point, with incurable and stigmatised patients (Butler, 1986; Fraser & Hesse, 1988).

Scholars from many fields have emphasised the importance of language in shaping public and political responses to changing practices in nursing. Definitions of ‘care’ and ‘caring’—terms with a deep but ambiguous history in nursing discourse—have come under scrutiny for their policy impact and implications (Antrobus, 1997; Woodward, 1997). In the NHS era, and particularly in the past quarter century, political and economic expediency has further muddied the waters by, for example, the 1999 decision to formally differentiate ‘nursing care’ and ‘personal care’ in order to means‐test access to the latter (Sweet with Dougall, 2008). While this distinction reflected a much longer trajectory of professionalisation and specialisation in the history of nursing, it also fixed a previously fluid boundary.

Considerable scholarly attention has focused on the paradox that nurse professionalism was, for much of its history, defined not by autonomy and exclusive knowledge (long seen as key to the status of doctors), but by obedience and practical experience (Rafferty, 1996). Beginning in the late 1960s, British structural and educational reforms, intended to redefine nursing professionalism along the lines followed by medicine, engendered significant tensions. For instance, writing in the wake of the 1976‐9 Royal Commission on the NHS, a prominent medical think tank, the King Edward's Hospital Fund (now the King's Fund) commented: ‘In nursing, there is a marked sense of bewilderment which derives partly from recent changes in clinical practice with consequent effects upon the nurse‐patient relationship. Above all, however, this uncertainty seems to stem from recent measures which have the effect … of detaching them from their patients and compelling the best of them towards a career in management’ (The King's Fund, 1977, p. 13). Acknowledging the ‘enthusiasm’ with which many nurses responded to greater opportunities for management and professional advancement, these unidentified professional observers nonetheless spoke of ‘a considerable sense of disquiet’ that ‘what Nightingale would have called “the art of nursing”—the giving of first place to the active care of the sick’ was being lost (The King's Fund, 1977, pp. 13–14). Others spoke about a ‘core of concern about people’ that was instilled into British nurses through their training, with its focus on ‘personal health care’, albeit largely in hospital and acute settings. Tellingly, this was positioned against the challenge ‘to become an “expert”’ in new technologies and clinical knowledge (The King's Fund, 1979, p. 2). The fact that such concerns were so often framed in comparison with a highly gendered historical past illustrates a key problem faced by all analyses of care and compassion in nursing: the abiding power of nostalgia for the ‘holistic’, vocational ‘basic nursing’ of the past. Reference to a ‘golden age’ of nursing and a desire to return to an idealised era appears to emerge in times of uncertainty, saying more about contemporary rather than past situations, and acting as a defence against current anxiety and as a barrier to change (Gillett, 2014).

By 1974, as the NHS moved from what was in retrospect a period of remarkable organisational stability to one of ‘continuous revolution’ (Webster, 1998, p. 140), the role, career structure and morale of nurses became a focus of intense discussion, particularly in the urgent and acute (hospital) care sector. In this period of growing economic crisis, labour unrest and rapid social change, debates over the future of nursing contained few explicit references to compassion as a professional desideratum. Observers of Britain's nursing profession instead commented on and generally deprecated what was perceived as a growing division between ‘direct care’ or ‘clinical nursing’ and ‘indirect care’ via technology, nursing management and education (Bradshaw, 2001; McKenna, Thompson, Watson, & Norman, 2006). Tellingly, the latter were seen as routes towards promotion and improved conditions and pay for nurses (Briggs, 1972; The King's Fund, 1977).

Some accounts from this period suggest scepticism about the idealised, maternally caring and compassionate nurse. For example, in a 1971 guide (Boorer, Craig, & Kirkpatrick, 1971) intended to help nurses critically consider their own attitudes towards patients, David Boorer (a public relations officer for the Confederation of Health Service Employees trade union), Janet Craig (then Director of the King's Fund Hospital Centre) and Bill Kirkpatrick (Assistant Regional Nursing Officer of London's North West Metropolitan Regional Hospital Board) proposed asking nurses to consider how their own attitudes were ‘influenced by regimes designed for the monasteries and convents of medieval times, and the fact that modern British nursing owes its foundation to a nineteenth century model of military medical care’. While the authors themselves took no position on these questions and were equally keen to challenge the ‘disease’ and ‘specialist’ attitudes ‘wherein [patients] are no longer regarded as people with individual … attitudes but rather as … cases’, their approach was explicitly intended to challenge normative assumptions about nurses as ‘natural’ carers. ‘How real’, they asked, ‘is our concern for the “person” of the patient? What lies behind our attitudes of “tender loving care”’ and the ‘conforming, infantile dependency’ that this reciprocally demanded from vulnerable patients? (Boorer et al., 1971, p. 3).

With the change of ideological regime marked by the 1979 election of Margaret Thatcher came a new set of responses to the NHS in general, and nursing in particular. The controversial Griffiths (1983) Report, which recommended the implementation of ‘general management’ (as opposed to management by clinically trained professionals) in the NHS—and, consequently, challenged the hard‐won autonomy, managerial responsibility and increased remuneration won by nurses in the previous decade—was presented by its proponents as ‘an opportunity to re‐professionalise nursing and … encourage a re‐evaluation of clinical nursing’ (Cole, 1987, p. 51). Yet in thus defining a need to ‘re‐professionalise nursing’, Griffiths and subsequent commentators implied that empowering (and paying) nurses to specialise or move into management devalued and deprofessionalised their activities on the wards: ‘Pre‐Griffiths … those nurses who enjoyed direct clinical contact were obliged to leave it for enhanced pay or status. Those who chose to remain were considered unambitious. Nurses reported feeling forced into management’ (Cole, 1987, p. 51). Commentators from within the nursing profession interpreted this rejection of the changes of the 1970s as a reaction against the increased status and cost of nurse–managers and highly trained specialist nurses, and against growing labour activism by state‐enrolled nurses and unqualified carers. Nurses argued against ‘the dilution of trained staff with untrained labour’ (explicitly looking back to the pre‐Nightingale era), and complained that the ‘female image’ and ‘dedication’ of nursing was ‘exploited by the authorities to ensure a depressed social status for nurses and a low level of pay’ (The King's Fund, 1984, p. 1).

In the increasingly marketised NHS of the 1980s, caring looked, at least to some nurses, like a futile effort. Yet discourse around compassion did begin to emerge in this context, particularly in relation to debates about nurse training, and the growing educational and professional divide between state‐enrolled and state‐registered nurses. Echoing today's debates about university‐trained nurses being ‘too clever to care’ (Gallagher, 2005, p. 14), nursing and external commentators looking back from the 1980s across the first three decades of the NHS bickered over the standards and outcomes of nurse training on nursing recruitment and practice. Were calls for higher educational levels among entrants to training ‘a manifestation of status seeking’ by the nursing authorities? Or was state resistance to such calls in fact simply an effort to economise on nursing costs and maximise nurse recruitment to the fledgling NHS, rather than a recognition of the fundamental importance to nursing of ‘personality traits’ sympathetic to the work of caring? Would a ‘better education’ really ‘preclude compassion? Do those without this education have a monopoly on compassion?’ (The King's Fund, 1984, p. 3). These are debates that have continued into the current era of the NHS, and are often played out within the popular press (Gillett, 2014).

## 3 CURRENT DEBATES AROUND COMPASSION IN HEALTHCARE

Today there is an extensive and international discourse, most prominent in bioethics, health services, nursing and medical research journals, but also visible in medical anthropology, sociology and history, concerned with questions of compassion and care in health professions. While doctors and nurses are identified as the key actors and vectors of compassion in these documents, it is telling that compassion appears as a fundamental organising principle of professional practice predominantly in relation to nursing.

In the light of evidence from the initial independent inquiry by Robert Francis (2010), NHS England (2012) published a 3‐year strategy for nurses, midwives and care staff on the delivery of high‐quality care, called ‘Compassion in Practice’. Six core values—care, compassion, competence, communication, courage and commitment—underpin this document, which states that compassionate care should also encompass how staff treat one another, because when feeling cared about it is easier to transfer this to the care of patients. Three years on from the launch of this strategy, an evaluation reported it had been implemented in a range of innovative ways, with organisational culture seen as crucial in the success of initiatives to promote and sustain compassionate care (Serrant, 2016). Yet recent research involving nursing, midwifery and care staff found that this policy is something that features in the working lives of senior staff but is outside the awareness of those attending directly to patients’ needs (O'Driscoll, Allan, Liu, Corbett, & Serrant, 2018); the study reported that this policy was regarded by participating nurses and midwives as a top‐down initiative, which failed to acknowledge the structural barriers they faced (e.g., short staffing, lack of resources) that affected their ability to provide compassionate care. This chimes with research by the authors to understand the meaning of compassionate care from the perspective of healthcare professionals working with patients who had type 2 diabetes (Tierney, Seers, Reeve, & Tutton, 2017). During data collection, between May and October 2015, none of the 36 participants mentioned ‘Compassion in Practice’ at any point during interviews and focus groups. They did, however, emphasise the need for structural and interpersonal factors to be considered when exploring compassionate care, rather than focusing on and blaming individuals (Tierney, Seers, Tutton, et al., 2017).

A recommendation in the Francis Report (2013, p. 1357) was for a ‘positive, safety culture’, reinforced by strong leadership that is steered by, among other values, compassion. Hence, the Francis Report (2013), and responses to it by the Royal College of Nursing (RCN, 2013), show that compassionate care is not just about individuals’ behaviour but the organisational culture within which they work (Seager, 2014). Nevertheless, research suggests there is a tendency to adopt an individual focus in discourse around compassionate care (Crawford, Brown, Kvangarsnes, & Gilbert, 2014; Tierney, Seers, Tutton, et al., 2017). This is reflected in a scoping study on the management of poor performance among nurses and midwives which, although not focused on compassionate care, highlighted how individuals within a healthcare system can be blamed and cast as a ‘bad apple’ (Traynor, Stone, Cook, Gould, & Maben, 2014, p. 55), when in fact failings are at a system level. The same could be said of a healthcare professional who is labelled as uncompassionate, with staff regarding this solely as a default of the individual rather than contemplating wider organisational issues requiring strong leadership to resolve (West, Eckert, Collins, & Chowla, 2017).

Individualising poor performance rather than adopting a broader systems level approach is reflected in the document ‘Delivering high quality, effective, compassionate care: Developing the right people with the right skills and the right values’ (Department of Health, 2013). It highlights the importance of educating staff to improve patient experiences and to ensure high‐quality, safe care. Following on from this publication, Values Based Recruitment has been incorporated into the selection of health professionals (Health Education England, 2016); students and staff are assessed on whether they are judged to exhibit values in line with the NHS Constitution, of which compassion is one (Department of Health, 2015). Yet focusing too much on individuals overlooks systemic challenges threatening compassionate care (Tierney, Seers, Tutton, et al., 2017), including staff shortages. As part of a response to these shortages, nursing associates have been introduced into the NHS. These posts are described as offering healthcare assistants the opportunity to move into a nursing role. However, a Health Foundation (2016) publication cautions that nursing associates should not be seen as a quick fix to staffing levels as it will take time for the job to become established. Likewise, the RCN has warned that these employees must not be used as a cheap replacement of registered nurses (Metcalf, 2017). What their introduction means for the delivery of compassionate care is not clear at present, but if registered nurses move away from the bedside, they may not be as involved in relational aspects of the job. The implication may be that registered nurses do not have the same opportunity to connect with patients on a human level, said to be a key part of providing compassionate care (Dewar & Nolan, 2013; Lown, Rosen, & Marttila, 2011).

Recent literature (Cole‐King & Gilbert, 2014; Maben, 2014) underscores how compassionate care can be demanding for staff practising in an environment where the overriding intention is to improve people's health, yet external demands are placed upon them, such as reaching targets and saving on costs (Tierney, Seers, Tutton, et al., 2017). It is proposed that when market forces predominate within health, it can result in care ‘being envisaged as a set of auditable transactions…instead of a covenant of care between caregiver and care receiver…’ (Iles, 2014, p. 188). For Borgstrom and Walter (2015, p. 103), a paradox is seen ‘between policy rhetoric about care being compassionate, and the reality of the commodification of care in which “presence” is the one thing that cannot be commodified…care packages need to include time for “presence” as well as time to do bodywork. In other words, caring about, as well as caring for, needs to be explicitly and financially recognised’.

## 4 IMPLICATIONS FOR NURSING

Although intermittently present within historical discourses, as alluded to above, compassion has attracted attention recently, both as a value and as a frame for practice in health and social care settings. It is painted as cost‐neutral in economic terms, and something that nurses are expected to express, as a consequence of their vocation or as a product of their training. Its cost‐neutrality has been questioned (Bivins, Tierney, & Seers, 2017) and risks overlooking an organisation's responsibility and role in the delivery of compassionate care, which can be emotionally draining, resulting in burnout without appropriate support and the risk of staff leaving their profession (Heinen et al., 2013; Maben, Latter, & Clark, 2007; McVicar, 2016). When unable to deliver care in a way they would wish, due to external pressures, health professionals can encounter ‘moral distress’ (Mauno, Ruokolainen, Kinnunen, & De Bloom, 2016), undertaking their work in an efficient manner but not connecting with patients, so that even those receiving first‐rate treatment do not feel cared about (Lee, 2016).

It has been noted that compassion requires a facilitative environment to flourish (Tierney, Seers, Tutton, et al., 2017). As alluded to above, policy documents have been criticised for painting compassion as a simple solution to recent problems identified in healthcare and for directing blame exclusively towards individuals (Crawford et al., 2014). When this is the case, there is the danger of overlooking how a system can also be at fault, meaning that cultural or organisational factors remain ignored and unchanged. Therefore, organisations need to consider how they are supporting or enabling the delivery of compassionate care. This includes nursing leaders, who through promoting a compassionate culture, in which staff feel psychologically safe, able to take appropriate risks, and are open to change, can contribute to innovation in healthcare (West et al., 2017).

## 5 CONCLUSION

Historically, professionals, politicians and the media have called for compassionate care most vocally when health services are perceived to be in crisis. This historical overview has outlined ways in which compassion is presented as a solution and painted in stereotypical terms, as something that is cost‐neutral and intrinsic to nursing. Categorising it in this way fails to acknowledge the emotional and practical resources required to make compassionate care a reality; it overlooks the role everyone working in healthcare, including managers and leaders, plays in reaching this goal, and risks reducing the provision of compassionate care to a task completed by individuals rather than an ethos to be embraced and supported by an organisation. A greater emphasis needs to be given to systems in which health professionals work and whether they support or hinder individuals in providing compassionate care.
